# Macromolecular biosynthetic parameters and metabolic profile in different life stages of *Leishmania braziliensis*: Amastigotes as a functionally less active stage

**DOI:** 10.1371/journal.pone.0180532

**Published:** 2017-07-25

**Authors:** Marlene Jara, Maya Berg, Guy Caljon, Geraldine de Muylder, Bart Cuypers, Denis Castillo, Ilse Maes, María del Carmen Orozco, Manu Vanaerschot, Jean-Claude Dujardin, Jorge Arevalo

**Affiliations:** 1 Instituto de Medicina Tropical “Alexander von Humboldt”, Universidad Peruana Cayetano Heredia, Lima, Perú; 2 Institute of Tropical Medicine Antwerp, Molecular Parasitology Unit, Antwerp, Belgium; 3 University of Antwerp, Antwerp, Belgium; 4 Laboratorios de Investigación y Desarrollo, Facultad de Ciencias y Filosofía, Universidad Peruana Cayetano Heredia, Lima, Perú; University of Ostrava, CZECH REPUBLIC

## Abstract

It was recently hypothesized that *Leishmania* amastigotes could constitute a semi-quiescent stage characterized by low replication and reduced metabolic activity. This concept developed with *Leishmania (Leishmania)* mexicana and *Leishmania (Leishmania) major* models might explain numerous clinical and sub-clinical features of *Leishmania (Viannia) braziliensis* infections, like reactivation of the disease, non-response to chemotherapy or asymptomatic infections. We compared here *in vitro* the proliferative capability of *L*. *(V*.*) braziliensis* amastigotes and promastigotes, assessed the expression of key molecular parameters and performed metabolomic analysis. We found that contrary to the highly proliferative promastigotes, amastigotes (axenic and intracellular) do not show evidence of extensive proliferation. In parallel, amastigotes showed a significant decrease of (i) the kDNA mini-circle abundance, (ii) the intracellular ATP level, (iii) the ribosomal components: rRNA subunits 18S and 28S α and ribosomal proteins RPS15 and RPL19, (iv) total RNA and protein levels. An untargeted metabolomic study identified clear differences between the different life stages: in comparison to logarithmic promastigotes, axenic amastigotes showed (a) a strong decrease of 14 essential and non-essential amino acids and eight metabolites involved in polyamine synthesis, (b) extensive changes in the phospholipids composition and (c) increased levels of several endogenous and exogenous sterols. Altogether, our results show that *L*. *(V*.*) braziliensis* amastigotes can show a phenotype with negligible rate of proliferation, a lower capacity of biosynthesis, a reduced bio-energetic level and a strongly altered metabolism. Our results pave the way for further exploration of quiescence among amastigotes of this species.

## Introduction

*Leishmania* is a Protozoan parasite with a life cycle involving invertebrate and vertebrate hosts: extracellular promastigotes proliferate in the sand fly mid-gut, while intracellular amastigotes are adapted to live within the phagolysosomes of mammalian macrophages [1]. In the human host, amastigotes cause a spectrum of clinical and sub-clinical manifestations including (i) asymptomatic infections, (ii) cutaneous leishmaniasis (CL), localized or diffuse, (iii) mucosal leishmaniasis (ML) with destructive mucosal inflammation of the oral and upper respiratory tract, (iv) visceral leishmaniasis (VL) with lethal dissemination to organs, or (v) post kala-azar dermal leishmaniasis (PKDL), a complication of VL [2].

Chronic persistence of parasites is a striking feature of *Leishmania* infections. This is among others illustrated by the presence of viable amastigotes in scars of CL lesions after therapy or even years after clinical cure [3–6] that eventually could produce reactivation of the lesion [7]. Immunological factors likely play a major role in this phenomenon, suggesting that parasites may persist for considerable periods of time until reactivation. There are few experimental studies on the physiology of the *Leishmania* amastigote stage, despite its clinical relevance, even less as for *L*. *(Viannia) braziliensis*, one of the most aggressive and prevalent species in the New World. Recent studies using heavy water labeling showed that the differentiation from promastigote of *Leishmania (Leishmania) mexicana* to amastigote was accompanied by cell duplication time increase, from 9 hours in cultured promastigotes to 4 days for *in vitro* intracellular amastigotes and 12 days for amastigotes isolated from lesions [8,9]. Moreover lesion-derived amastigotes of *L*. *(L*.*) mexicana* showed evidence of significantly lower rates of RNA synthesis, protein turnover and membrane lipid synthesis than *in vitro* amastigotes [8]. Accordingly, Kloehn and collaborators hypothesized that amastigotes would live in a semi-quiescent physiological state *in vivo*. A recent report on *Leishmania (Leishmania) major* showed that the picture could be even more complex, with the co-existence of ‘fast’ (60 hours doubling time) and low/non-replicating amastigotes in infected mice [10] but the authors could not assess the metabolic status of these different types of parasites.

Quiescence is a common mechanism of persistence for infectious microorganisms, including bacteria like *Mycobacterium tuberculosis* and *Staphylococcus aureus* and protozoa like *Plasmodium vivax* and *Babesia* sp. [11,12]. The quiescent form is defined as a reversible non-proliferative or low proliferative stage with reduced metabolic activity. Quiescent cells often represent only a small fraction of the original whole population and are produced in response to starvation or environmental stress (host immune response, drug pressure), providing a mechanism for pathogens to persist for months or even years and avoid population extinction [5,13].

In order to start exploring *in vitro* the possible existence of quiescence in in *L*. *(V*.*) braziliensis*, a highly relevant but challenging model, we evaluated the kinetic of proliferation and quantified specific macromolecules and metabolic indicators of the biosynthetic and energetic level. Our results show that *in vitro*, *L*. *(V*.*) braziliensis* amastigotes can show a phenotype with negligible rate of proliferation, a lower capacity of biosynthesis, a reduced bio-energetic level and a strongly altered metabolism. Our results pave the way for further exploration of quiescence in this species.

## Materials and methods

### Ethics statement

Mice were used for isolation of peritoneal macrophages by methods approved by the ethical committee from the Institute of Tropical Medicine (Antwerp). They certified that Protocol N° MPU2014-2 follows the guidelines of the Federation of European Laboratory Animal Science Associations (FELASA). The female BALB/c mice (6–8 weeks of age) were sacrificed in a CO2 chamber.

### Culture of promastigotes

The reference strain *L*. *(V*.*) braziliensis* MHOM/BR/75/M2904 was cultivated in M199 medium supplemented with 20% Fetal Bovine Serum (FBS), 100 units/mL of penicillin and 100 μg/mL of streptomycin. A growth curve of *Leishmania* promastigotes was done with an initial concentration of 0.5 x 10^6^ parasites/mL. The parasites were fixed with 2% paraformaldehyde in PBS and counted daily for 6 days in a hemocytometer. In the next paragraphs the abbreviations Pro^log^ and Pro^stat^ will be used for logarithmic and stationary promastigotes, respectively.

### Culture of axenic amastigotes and intracellular amastigotes

In order to obtain axenic amastigotes, promastigotes (1.5x10^6^ parasites/mL) were maintained in complete M199 medium (M199 medium supplemented with 20% FBS, 100 units/mL penicillin and 100 μg/mL of streptomycin) at pH 7.2 at 25°C. On the fourth day, promastigotes were centrifuged (1500 g) and the pellet was resuspended in complete M199 at pH 5.5 and incubated at 34°C. After four days, the resulting axenic amastigotes (Ama^axe^) were harvested or used to infect murine peritoneal macrophages at a ratio of 4 or 8 Ama^axe^ to 1 macrophage in flasks of 75 cm^2^, in triplicate. Infected macrophages were incubated at 34°C in a 5% CO_2_/air mix for 72 hours [14]. In order to ensure that the results of the proliferation assay would not be influenced by a lower virulence of our reference strain M2904, we infected macrophages with a clinical isolate of *L*. *(V*.*) braziliensis*, MHOM/PE/03/PER206. For rRNA and kDNA quantifications, intracellular amastigotes (Ama^int^) were co-harvested with macrophages at 72 hours post-infection using a protocol reported elsewhere [14,15]. For the measurements of the ADP/ATP ratio and the quantification of RPL19 and RPS15, Ama^int^ and Ama^axe^ were purified by centrifugation in a percoll gradient as reported elsewhere [16]. In parallel, infection rates of peritoneal macrophages were monitored using 16-well chamber slides as a control; 100 cells were counted in order to determine the percentage of infected macrophages and the average number of amastigotes per infected macrophage. Whenever technically possible, all assays were applied on both types of amastigotes (Ama^axe^ and Ama^int^), but for some of them, only Ama^axe^ were accessible to the analyses.

### Viability assay

Cell viability was assessed using the cell membrane non-permeable stain NucGreen (Thermo fisher scientific), that emits bright green fluorescence when bound to DNA on cells with membrane damage, and the cell membrane permeable stain Vybrant DyeCycle^™^ Ruby stain (Thermo fisher scientific) that stains the nucleus and cytoplasm of whole cells. Briefly, a 3X solution of each stain was prepared by mixing 1.5 μl of the Vybrant DyeCycle and 3 drops of the NucGreen per mL of PBS. Then, 100 μL of each 3X solution was added to 100 μL of parasites at a density of 1.5 x 10^6^/mL and incubated at 37°C for 15 min before being analyzed with the BD FACSVerse^™^ Flow Cytometer. Unstained and single stained controls were included in each experiment and analysis of the flow cytometric data was performed using the FCS express 5 Plus Research Edition.

### Quantification of the total protein and total RNA content

The whole RNA (5 x10^7^ promastigotes and Ama^axe^) was isolated with the Trizol LS reagent (Invitrogen), according to the manufacturer’s instructions. The isolated RNA was quantified by fluorometry using the Quant-iT^™^ BR RNA Assay Kit and the Qubit fluorometer (Invitrogen). The interphase generated after the separation step of the RNA isolation protocol was stored at -70°C for posterior DNA isolation. The RNA samples were stored at -70°C until subsequent cDNA synthesis. For the total protein content quantification, 5x10^7^ parasites were harvested and re-suspended in 500 μL of Lysis buffer (150 mM NaCl, 1% Triton X-100 and 50 mM Tris/HCl), containing cOmplete^™^, mini protease inhibitor (Roche). This suspension was incubated for 30 min at 4°C and then sonicated for 2 min. The total proteins in this crude extract were quantified with the *RC DC*^*™*^ protein assay kit (Biorad). Proteins were stored at -70°C until western blot analysis. The total content of proteins and RNA were expressed as μg / 10^6^ cells.

### Relative quantification of the kDNA minicircles

Relative quantification of the kDNA minicircles was performed for promastigotes, Ama^axe^ and Ama^int^ by using two quantitative real time PCR assays (qPCR) targeting the kDNA mini-circle [17] and the single copy gene G6PD [18]. The gene from G6PD is located on chromosome 20, shown to be disomic in most of the *L*. *(V*.*) braziliensis* strains we sequenced (unpublished results). Accordingly, the aneuploidy often described in *Leishmania* should not interfere with the quantification process [19]. The DNA isolated from the interphase after RNA isolation with Trizol (see above) was used here. We included three technical replicates per sample and three biological replicates per stage. The relative variability of the number of copies of kDNA minicircle was calculated as follows: (i) parasite DNA equivalent per reaction, estimated by kDNA qPCR divided by (ii) parasites per reaction estimated by G6PD qPCR. As the number of copies of G6PD per parasite is constant, deviation on the estimation of the number of parasites between the qPCR kDNA and the qPCR G6PD reflect changes in the number of copies of kDNA minicircles. These ratios were then rescaled in comparison to the Pro^log^.

### Quantification of ribosomal RNA 18S and 28S α

The cDNA of promastigotes and amastigotes was synthesized with 200 ng of RNA using the Transcriptor First Strand cDNA Synthesis Kit (Roche) and the reverse primers for rRNA 18S and 28S α subunits according to manufacturer’s instructions. The sequence of the primers for the cDNA synthesis and qPCR are described in S1 Table.

Two SYBR Green-based real-time quantitative PCR (qPCR) assays were developed for absolute quantification of *L*. *(V*.*) braziliensis* cDNA corresponding to the rRNAs 18S, and 28S α. The qPCR reactions were performed in a 25 μL reaction mixture consisting of 5 μL cDNA, 300 nM of each primer (S1 Table), and 1X iQ^™^ SYBR Green Supermix (Bio-Rad). Reactions were run on the LightCycler 480 system (Roche). The thermal cycling conditions were as follows: 95°C for 3 min, 35 cycles at 95°C for 20 s, 60°C for 20 s and 72°C for 20 s. Fluorescence emission was measured at the end of the elongation step. After PCR amplification, a melting curve was generated for every PCR product to check the specificity of the PCR reaction (absence of primer-dimers or other nonspecific amplification products). The melting curve analysis consisted of 1 cycle at 95°C for 60 s, followed by 40°C for 60 s and continuous heating at 0.02°C/s to 95°C. Data were collected and analyzed with the LightCycler TM software v1.5.0. Each run included a positive control sample (DNA from promastigotes of strain M2904), a negative control (mix of reverse transcription without reverse transcriptase) and a blank (no-template control). Each sample was tested in triplicate. The threshold cycle (Ct) values of triplicate measurements were averaged.

The standard curves for the qPCR assays were generated using 10-fold serial dilutions of two pGEM-T Easy Vector (Promega) containing a sequence of the rRNA 18S and 28S α (PCR product) cloned from strain MHOM/BR/75/M2904 (S1 Fig). Under the standardized conditions both assays did not amplify genomic DNA or cDNA of mouse.

### Western blot for ribosomal proteins RPL19 and RPS15

For Western Blot, 20 μL of proteins (after the lysis of 5x10^7^ parasites in 240 μL of Triton X-100 buffer) were run in a SDS-PAGE electrophoresis. The proteins were transferred to a PVDF membrane with a tank blotting unit, during 1 hour at 100 V (250 mA). The membrane was blocked with T-TBS 5% nonfat dry milk, then immuno-probing of RPL19 was done with a commercial antibody (Biomatik) produced in goat against the synthetic peptide C- LRRYRESKKIDRH. This peptide has a sequence showing 100% Query cover, 100% percent of similarity and 81.8% of identity with respect to the sequences of RPL19 in the reported genome of *L*. *(V*.*) braziliensis* (NCBI). The detection was done using an anti-goat IgG produced in rabbit conjugated with horseradish peroxidase (IgG-HRP) (Abcam) and the Pierce ECL Western Blotting Substratee (Thermo scientific). Immunoblotting of RPS15 was performed with the commercial antibody (Antibodies-online) produced in rabbit against CGVYNGKTFNQVEIKPEMIGHYLGEFSITYKPVKHGRPGIGATHSSRFIPL a synthetic peptide. This peptide has a sequence showing 100% Query cover, 74% identity and 88% similarity with respect to the sequence of RPS15 in *L*. *(V*.*) braziliensis*. The detection was done using an anti-rabbit IgG-HRP produced in goat. After the western blot of the ribosomal proteins, the PVDF membranes were washed with a Restore^™^ Western Blot Stripping Buffer (Thermo scientific) in order to remove previous primary and secondary antibody and they were submitted to immunoblot with α-tubulin as loading control. The relative expression of RPL19 and RPS15 were calculated by densitometry and normalized to the expression of α-tubulin using the software Quantity One (Biorad). The relative expression of RPS15 and RPL19 were rescaled to the expression of Pro^log^.

### Quantification of ADP/ATP ratio

The ATP and ADP content in promastigotes and amastigotes of *Leishmania* was measured with the ADP/ATP Ratio Assay Kit (Sigma). This kit is based on the luminescent enzymatic reaction mediated by luciferase between (i) the ATP released from the cells after the lysis and (ii) the substrate D-luciferin. After ATP measurement, ADP is quantified by converting it to ATP which can be detected with the same luciferase assay. From here ADP/ATP RLU (Relative light unit) ratios and individual ATP RLUs and ADP RLUs were determined. Three biological replicates of each *Leishmania* life stage and three technical replicates per stage were assessed in a 96 wells white flat-bottom plate and processed according to the manufacturer’s instructions. The luminescence was quantified with the CHAMELEON^™^V microplate reader. A blank without cells was also included in triplicate; the mean luminescence in these wells (background) was subtracted from the luminescence from wells with samples before quantitative analysis.

### Untargeted metabolic profiling of promastigotes and axenic amastigotes

Samples of Pro^log^, Pro^sta^ and Ama^axe^ were quenched by rapid chilling (each stage had 4 biological replicates) and metabolites were extracted as reported elsewhere [20]. Samples were analyzed by Liquid Chromatography and Mass Spectrometry on an Exactive Orbitrap mass spectrometer (Thermo Fisher) at the Scottish Metabolomics Facility (Glasgow Polyomics, University of Glasgow) in both positive and negative modes (rapid switching), coupled to a U3000 RSLC HPLC (Dionex) with a 2.1 mm ZIC-HILIC column (Sequant) as described previously [21]. Sample list set up was performed as described in [21,22], data processing (including normalization to total ion chromatogram) and metabolite identification was performed in MzMatch.R as extensively described in [20,21]. Relative quantification was based on raw peak heights, and expressed relative to the (average) peak height of another line (Ama^axe^ versus Pro^log^ or Ama^axe^ versus Pro^sta^). This relative expression will hereafter be called the ‘fold change’. Metabolite changes were considered to be biologically significant when the ratio of signal intensity (normalized to total ion chromatogram) between 2 samples was higher than 2 (significant increase) with a t-test *p*-value < 0.05 or lower than 0.5 (significant decrease) with a t-test *p*-value < 0.05; other ratios were considered as non-significant changes. To check the consistency of the metabolic changes across the biological replicates, heatmaps were generated with the R package ‘gplots’ for several subsets of metabolites (amino acids, glycerophosphocholines and other glycerophospholipid). These heatmaps also allowed the simultaneous comparison of the 3 experimental conditions (Ama^axe^, Pro^log^ and Pro^sta^), while FCs allow only pairwise comparisons.

## Results

### Growth of promastigotes and amastigotes of *L*. *(V*.*) braziliensis*

**Fig 1:**
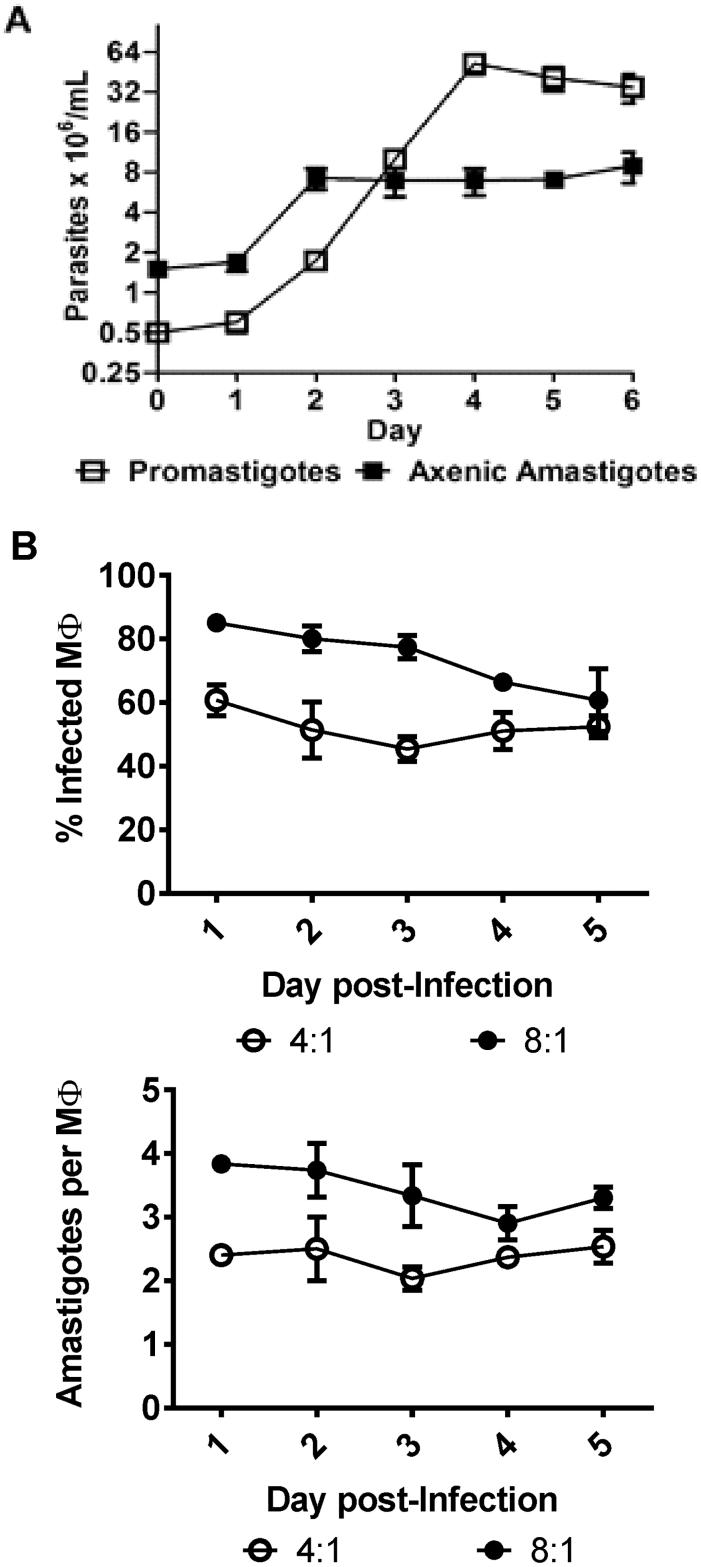
*L*. *(V*.*) braziliensis* growth kinetics. **A**, *In vitro* growth curve of promastigotes and axenic amastigotes. **B**, Kinetics of *in vitro* infection of Ama^int^ in macrophages, using 2 different infection ratios (4:1 and 8:1). The average of three biological replicates and standard errors of the means (SEM) are presented.

Promastigotes of the reference strain M2904 reached their stationary phase on the fourth day with a density of 52.3 x 10^6^ ± 5.0 parasites/mL (Mean ± SEM) of culture (Fig 1A). The mean duplication time for promastigotes during the logarithmic phase was 11.8 ± 2.0 hrs. For the next experiments, Pro^log^ were harvested on the third day, while Pro^sta^ were harvested on the sixth day. The evaluation of the growth of M2904 axenic amastigotes immediately after the transfer of early stationary promastigotes to acidic pH and increased temperature showed a stationary behavior since the second day with a density of 7.2 x 10^6^ ± 0.78 x 10^6^ (Mean ± SD). The *in vitro* infection kinetics of M2904 amastigotes in peritoneal macrophages from mice was evaluated over 5 days with two ratios of parasites: macrophages (Fig 1B). Using a 8:1 ratio, infection parameters were rather stable over the 5 days of culture, with a percentage of infected macrophages of 73.9 ± 4.5% and an average number of intracellular amastigotes per infected macrophage of 3.4 ± 0.2. Infection parameters were also stable with a 4:1 ratio, with 52.1 ± 2.5% of infected macrophages and 2.4 ± 0.1 amastigotes per infected macrophage. Thus, M2904 Ama^int^ did not show evidence of proliferation even in the availability of free macrophages. This result could be explained by a possible avirulence of M2904, a strain maintained for years in experimental conditions. To exclude this possibility, the intracellular infection of macrophages was repeated with a clinical isolate (PER206), that was maintained for a minimum of passages since isolation from a patient: as in the case of M2904, infection parameters were stable with a 8:1 ratio, with 65.0 ± 10.9% of infected macrophages and 3.1 ± 0.4 amastigotes per infected macrophage (S2 Fig). The very low proliferation encountered in Ama^axe^ and Ama^int^ of M2904 was also not due to similar rates of replication and cell death, as in that case a significant number of death cells should be encountered. This was verified by flow cytometry analysis of cells stained with permeable and non-permeable fluorophores: this showed a minimal proportion of death cells (0.6% in Ama^axe^ and 6.8% in Ama^int^ vs 1.1% in Pro^log^ (S3 Fig).

### ADP/ATP ratio during the life cycle

**Fig 2:**
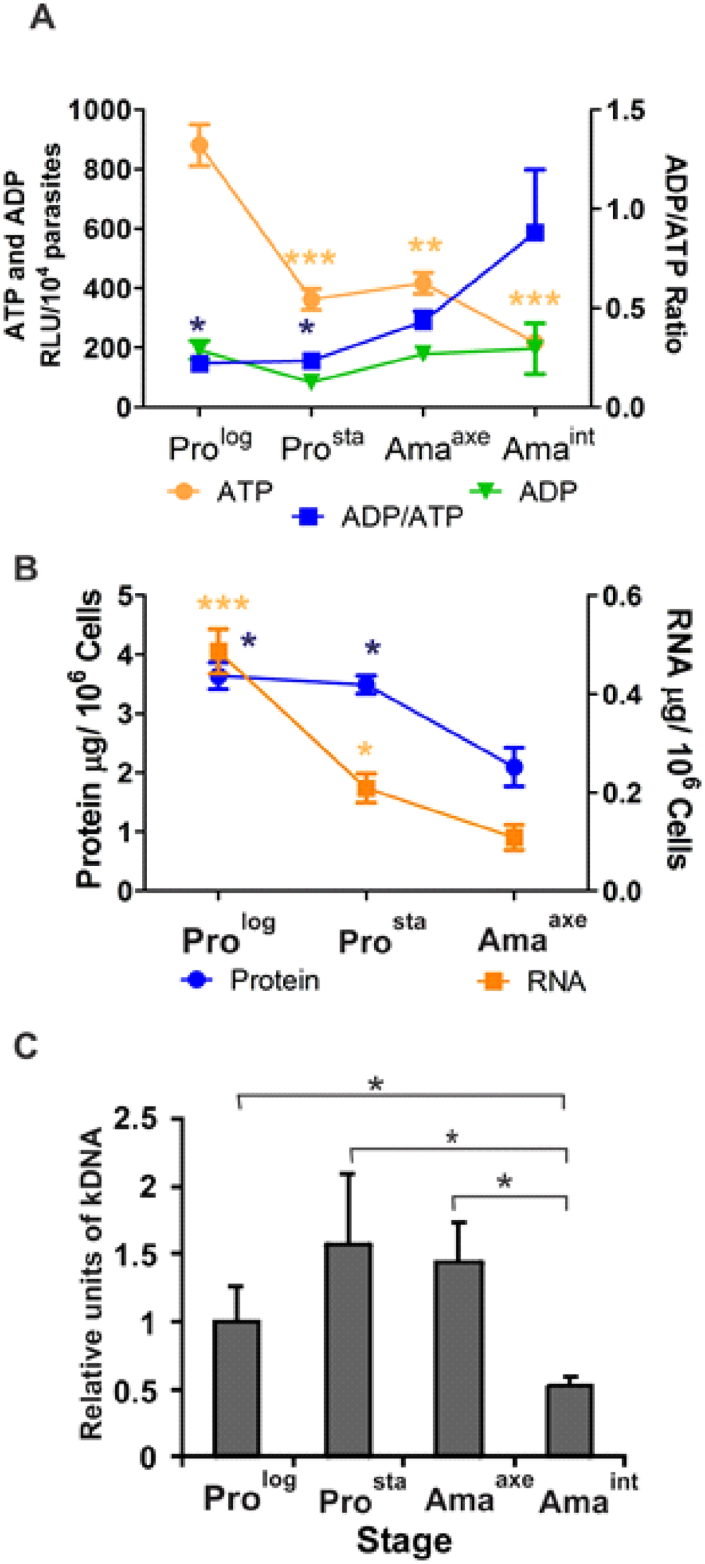
Quantification of ATP/ADP, total RNA, protein content and kDNA minicircles during the life cycle of *L (V*.*) braziliensis*. **A**, Ama^int^, Ama^axe^ and Pro^sta^ show decreased levels of ATP in comparison to Pro^log^. The asterisks represent statistically significant differences in comparison to Pro^log^. Because of the decreased levels of ATP, ADP/ATP ratio is increased in Ama^int^ in comparison to the Pro^log^ and Pro^sta^. The asterisks represent statistically significant differences in comparison to Ama^int^. **B**, The Ama^axe^ show a decrease in the total content of RNA and protein in comparison to Pro^log^ and Pro^sta^. The asterisks represent statistically significant differences in comparison to Ama^axe^. **C**, The kDNA mini circles decrease in Ama^int^ in comparison to Pro^log^, Pro^sta^ and Ama^axe^. The asterisks represent statistically significant differences after multiple t-test between all the pairs. The number of asterisks in figures A and B represents different significance levels (* P ≤ 0.05 and ** P ≤ 0.01) using one-way ANOVA followed by the Bonferroni's Multiple Comparison Test.

Proliferation and macromolecular biosynthetic processes require a high ATP consumption. Therefore, we quantified ATP with a luminescent enzymatic reaction, in order to assess the energy status of each *Leishmania* stage. The ATP-RLUs were 880 ± 70 (mean ± SEM), 360 ± 35, 420 ± 35 and 220 ± 18 for Pro^log^, Pro^sta^, Ama^axe^ and Ama^int^, respectively. Overall, the amount of ATP decreased significantly in Ama^int^ (P ≤ 0.001), Ama^axe^ (P ≤ 0.01) and Pro^sta^ (P ≤ 0.001) in comparison to Pro^log^ (One way ANOVA P = 0.0001, Bonferroni's multiple comparison test) (Fig 2A). The ADP-RLUs were 190 ± 31, 80 ± 10, 180 ± 27 and 200 ± 85 for Pro^log^, Pro^sta^, Ama^axe^ and Ama^int^, respectively: these differences were not significant (One way ANOVA P = 0.17). Essentially because of the decrease in ATP, the Ama^int^ had the highest ADP/ATP ratio (0.88 ± 0.32) in comparison to Pro^log^ (0.22 ± 0.04), Pro^sta^ (0.23 ± 0.03) and Ama^axe^ (0.43 ± 0.05). The differences were statistically significant between Ama^int^ and Pro^log^ (P≤0.05) and Pro^sta^ (P≤0.05) (One-way ANOVA P = 0.02, Bonferroni's multiple comparison test). Altogether, our results show that non-proliferative stages as Pro^sta^ and amastigotes have decreased levels of ATP in comparison to the proliferative Pro^log^.

### Quantification of total RNA, protein and kDNA during the life cycle

Ama^axe^ had a significantly lower amount of total RNA (0.111 ± 0.025 μg/ 10^6^ cells) than Pro^log^ (0.505 ± 0.045 μg/ 10^6^ cells) and Pro^sta^ (0.207 ± 0.029 μg/ 10^6^ cells) (One-way Anova, *P*<0.001, Bonferroni's multiple comparison test). Similarly, total protein content per cell was lower in Ama^axe^ (2.1 ± 0.57 μg / 10^6^ cells) than in Pro^log^ (3.64 ± 0.39 μg/ 10^6^ cells) and Pro^sta^ (3.49 ± 0.26 μg/ 10^6^ cells) (One-way Anova, *P* = 0.008, Bonferroni's multiple comparison test) (Fig 2B). The relative abundance of kDNA (minicircles) was assessed throughout the life cycle of the parasites (Fig 2C). This reached 1.0 ± 0.26 relative units (Mean ± SD) in the Pro^log^, 1.57 ± 0.51 in the Pro^sta^, 1.44 ± 0.41 in Ama^axe^ and 0.53 ± 0.06 in Ama^int^. Accordingly, *in vitro* Ama^int^ had significantly fewer copies of kDNA minicircles than Pro^log^ (t-test, *P* = 0.036), Pro^sta^ (t-test, *P* = 0.02) and Ama^axe^ (t-test, *P* = 0.006).

### Quantification of 18S and 28S α rRNA molecules during the life cycle

**Fig 3:**
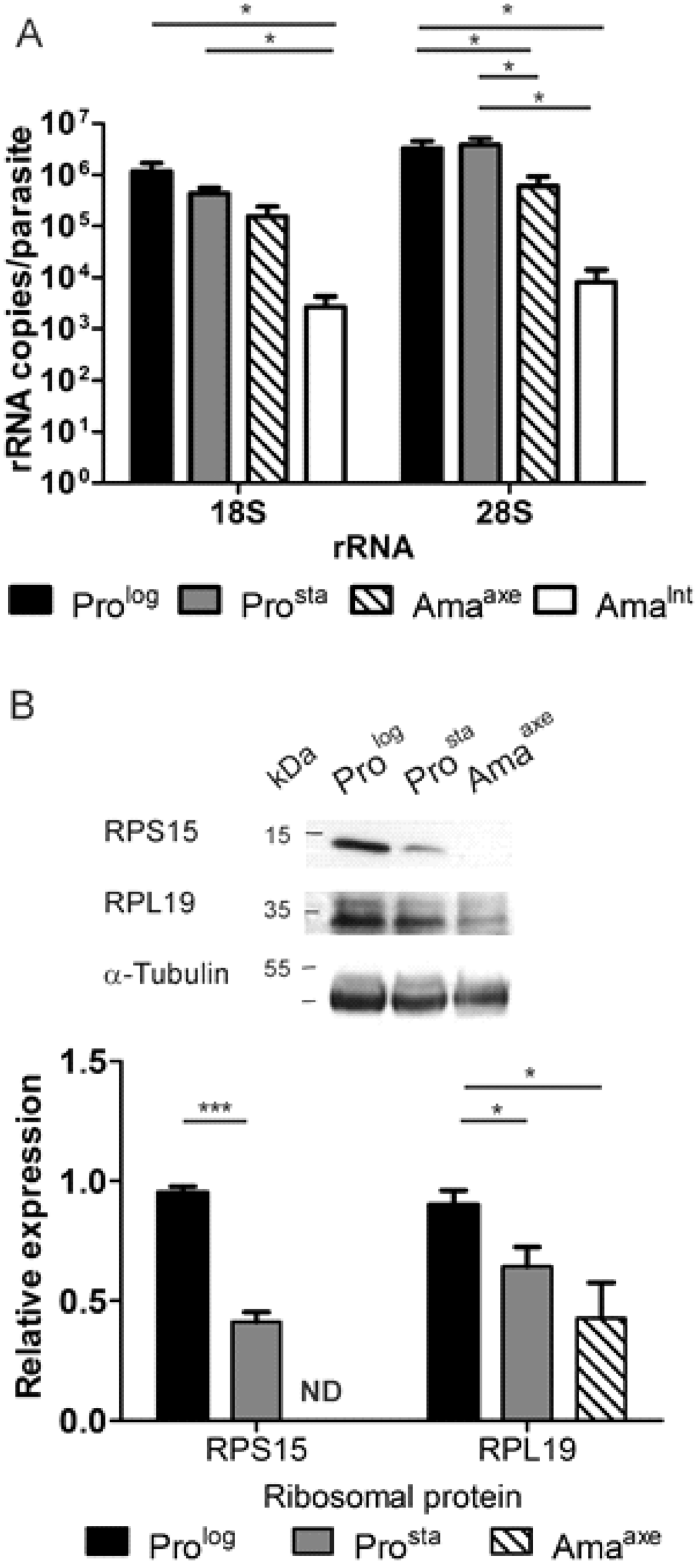
Decrease of rRNAs and ribosomal proteins in *L*. *(V*.*) braziliensis* amastigotes. **A**, Absolute quantification per parasite of rRNAs 18S and 28S α after reverse transcription and qPCR. The asterisks represent statistically significant differences between different pairs after performing multiple t-test. **B**, Evaluation of the expression of ribosomal proteins. The expression of RPS15 and RPL19 was normalized to the expression of α-tubulin and the results were rescaled to the expression on Pro^log^. The bars represent the mean ± SEM in three biological replicates. The number of asterisks represents different significance levels (* P ≤ 0.05 and ** P ≤ 0.01). ND, No bar is plotted because the expression of RPS15 in Ama^axe^ was below the detection limit.

The process of proliferation requires a high amount of proteins [23]. To probe the protein synthesis machinery, we analyzed ribosomal components in the different *Leishmania* stages. The copy number of 28S α rRNA per parasite (Fig 3A, right) was 331 ×10 ^4^ ± 120 × 10^4^ (mean ± SEM) for Pro^log^ and 380 ×10^4^ ± 124 × 10^4^ for Pro^sta^. This number decreased significantly to 59.6 × 10^4^ ± 32.2 × 10^4^ in Ama^axe^ (t-test, P = 0.047 and 0.033 vs Pro^log^ and Pro^sta^, respectively). In Ama^int^, this number decreased even further to 0.82 x 10^4^ ± 0.59 ×10^4^ (P = 0.026 and 0.046 vs Pro^log^ and Pro^sta^, respectively; P = 0.105 vs Ama^axe^; t-test).

A similar trend was observed with 18S rRNA (Fig 3A left), with an average copy number of 18S rRNA per parasite of 117.0 ×10^4^ ± 51.1 × 10^4^ and 42.7 × 10^4^ ± 12.4 × 10^4^ for Pro^log^ and Pro^sta^, respectively, and 15.4 × 10^4^ ± 8.7 × 10^4^ and 0.27 x 10^4^ ± 0.16 ×10^4^ for Ama^axe^ and Ama^int^, respectively: differences were just below the level of statistical significance between Ama^axe^ and promastigotes (0.059 and 0.072, vs Pro^log^ and Pro^sta^, respectively; t-test), while they were significant for Ama^int^ (respectively P = 0.042 and 0.038). The differences between Ama^int^ and Ama^axe^ did not reach statistical significance (t-test, P = 0.113). Our results thus show a decrease of rRNA molecules per parasite during the life cycle, with the lowest number in the Ama^int^.

### Evaluation of ribosomal proteins RPS15 and RPL19 expression by western blot

If the number of ribosomes varies during the lifecycle of *L*. *(V*.*) braziliensis*, the decrease in rRNA described here above should be accompanied by a decrease in the abundance of ribosomal proteins [24]. Therefore, we evaluated the expression of the ribosomal proteins RPS15 (small ribosomal subunit) and RPL19 (large ribosomal subunit) in promastigotes and Ama^axe^ (Fig 3B). The expression of RPS15 was the highest in Pro^log^ and decreased significantly in Pro^sta^ (from 0.95 ± 0.04 (Mean ± SD) to 0.41 ± 0.07; t-test, *P* = 0.0003). In Ama^axe^, it was under the limit of detection. Since three sequences of RPL19 are present in the genome of *L*. *(V*.*) braziliensis*, the identity of the RPL19 present in the ribosomes was corroborated by including a sample of polysomes isolated by a gradient of sucrose and ultracentrifugation (data not shown). Similarly to RPS15, the expression of RPL19 was highest in the Pro^log^ (0.9 ± 0.1) with a gradual and statistically significant decrease in the Pro^sta^ (0.64 ± 0.14; t-test, *P* = 0.036) and Ama^axe^ (0.43 ± 0.26; t-test, *P* = 0.02).

### Untargeted metabolic profiling of promastigotes and axenic amastigotes

Untargeted LC-MS metabolomics was performed on Pro^log^, Pro^sta^ and Ama^axe^ to characterize the metabolic changes occurring in the different life stages. In total, 186 metabolites were putatively identified with a mass accuracy of 2 ppm: glycerophospholipids (GPLs: 91), amino acids and derivatives (30), fatty acyls (8), precursors and derivatives of steroids (10), nucleobases and nucleosides (9), carbohydrates (6), sphingolipids and sphingoid bases (8), and other metabolites (24). More detailed information on the specific metabolites can be found in the supplementary data file (S2 Table).

**Fig 4:**
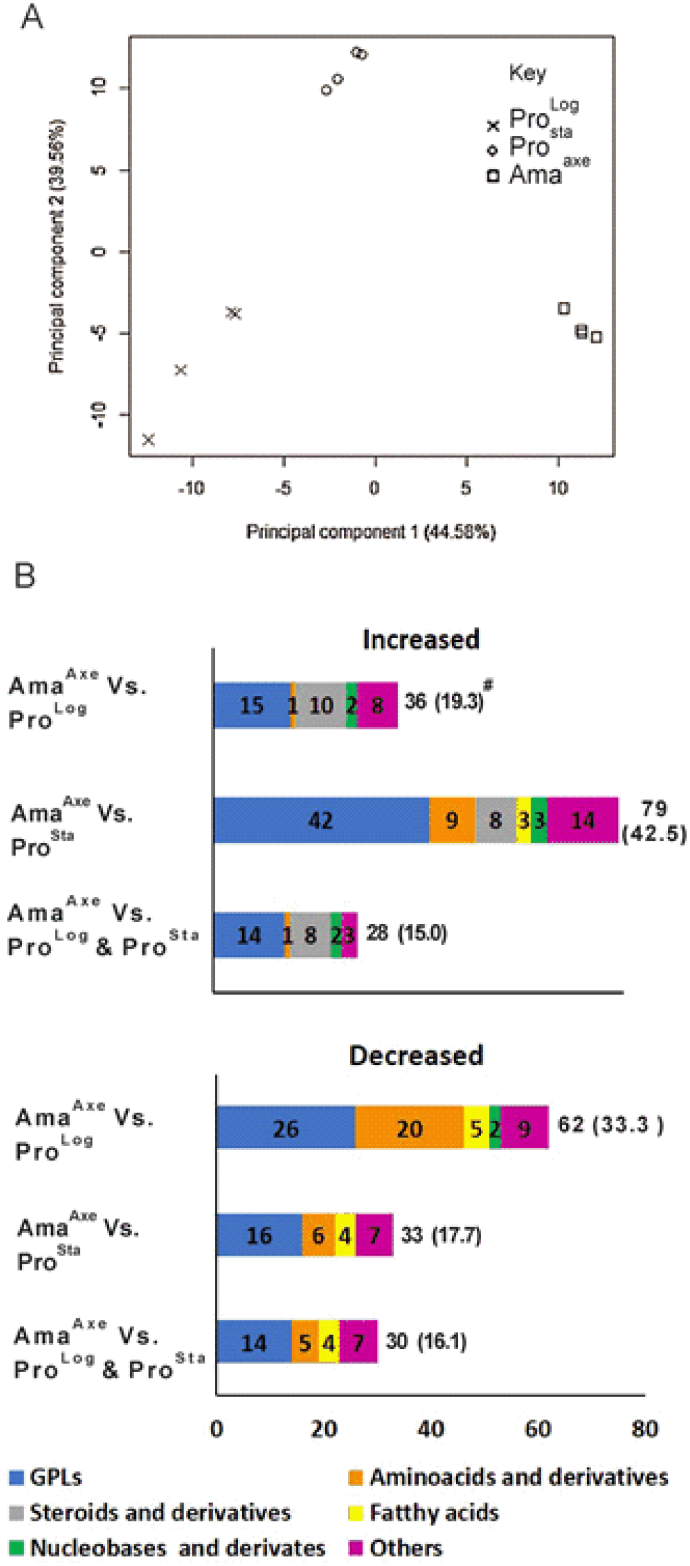
Metabolic profile in promastigotes and axenic amastigotes. **A**, Principal component analysis (PCA) distinguishing the metabolome of three growth stages in *L (V*.*) braziliensis*. PCA shows unsupervised clustering based on the quantitative information of all 186 putatively identified metabolites. **B**, Number of metabolites, organized by main metabolic pathways, with significantly increased (> 2 fold; t-test, P< 0.05) or decreased (<0.5 fold; t-test, P< 0.05) levels in Ama^axe^ in comparison to Pro^log^, Pro^sta^ or both. For each growth stage, four biological replicates were included. The detailed results of the 186 metabolites can be found in the supporting information (S2 Table). ^#^ Total number and percentage (calculated on the base of 186 identified metabolites).

Principal component analysis (PCA) summarizes most of the variation in the complete dataset in just a few principal components and thus enables the visualization of the major differences between samples in a simple 2-dimensional plot (Fig 4A). The first principal component (PC1: 44.58%) clearly separated the Pro^log^, Pro^sta^ and Ama^axe^, moreover PC1 showed that the highest variation was present between the samples from Pro^log^ and Ama^axe^. The second principal component (PC2: 39.56%) clearly separated the Pro^sta^ from Pro^log^ and Ama^axe^. Fig 4B summarizes the grouped metabolites that are significantly increased or decreased in Ama^axe^ versus Pro^log^ and Pro^sta^ (considered separately or together). This highlights among others that steroids and derivatives all increased in Ama^axe^, while amino acids were mostly in the category of decreased metabolites in this life stage.

**Fig 5:**
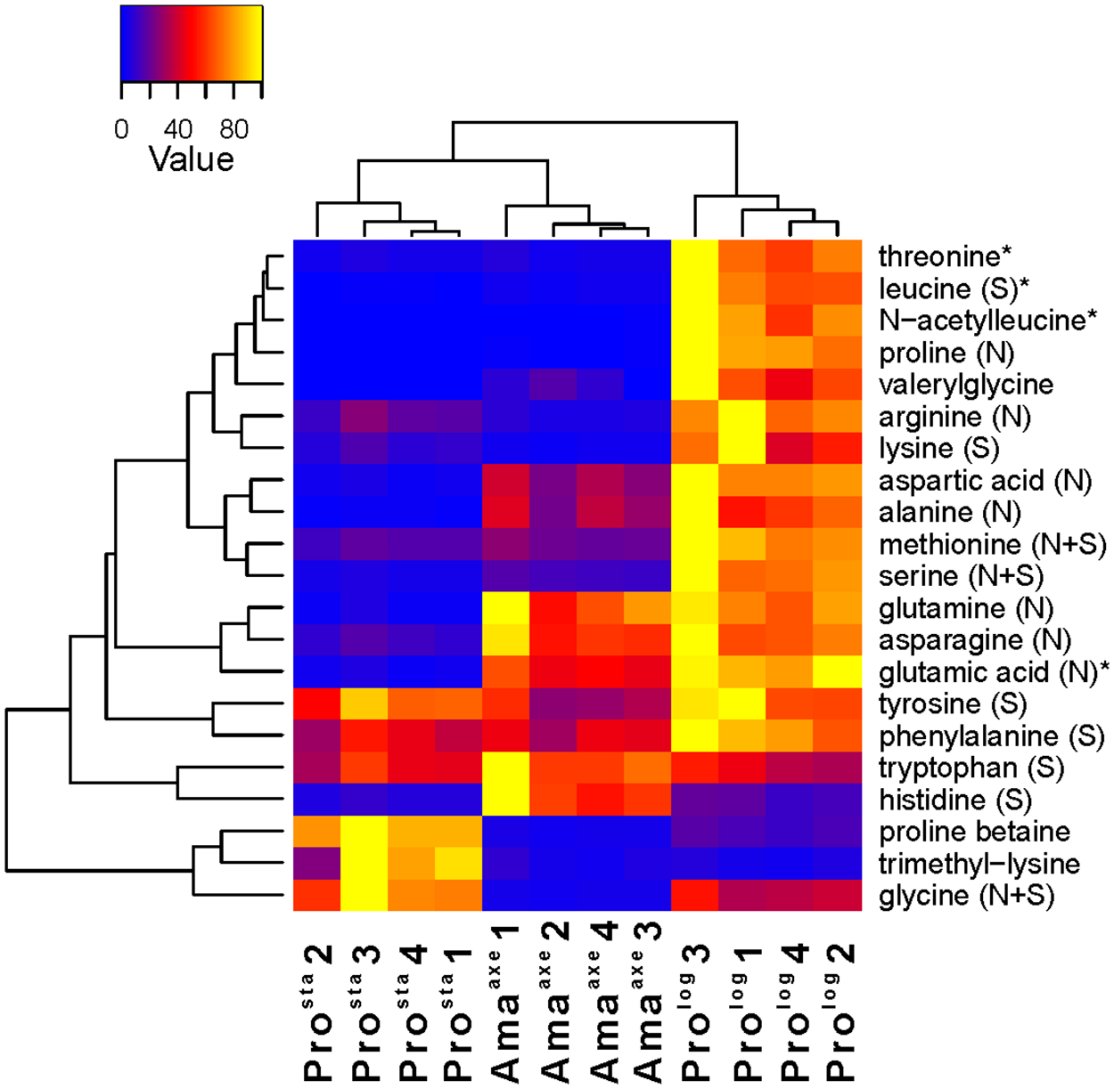
Metabolic profiles of amino acids and derivatives in heatmap format. The samples are presented along the bottom (Pro^sta^, Ama^axe^, Pro^log^, the numbering represent the biological replicate). For each metabolite, its abundance over the different biological replicates was rescaled between 0 (blue) and 100 (yellow). The corresponding fold changes between conditions are displayed in S3 Table. On the right, the amino acids and derivatives are represented (S: salvage (essential) amino acids, N: *de novo* (non-essential) amino acids, N+S: amino acids that can be both synthesized *de novo* and salvaged, but an incomplete pathway for *de novo* synthesis is described in the Leishcyc and KEGG metabolic databases). The asterisk indicates that another isomer was detected for this metabolite (see S2 Table for more details). The branches represent the dendrogram after hierarchical cluster analysis.

All the amino acids with the exception of cysteine were detected in the present study and they showed a different pattern in each stage (Fig 5 and S3 Table). A reduction of the levels of 14 amino acids (Fold <0.5; t-test, P<0.05) was shown in Ama^axe^ in comparison to Pro^log^ (in average, 0.2 fold), while only histidine showed a significant increase (Fold >2; t-test, P<0.05). When the Ama^axe^ were compared with the Pro^sta^, the former showed a decrease in the levels of 4 amino acids (Lys = 0.31, Arg = 0.35, Val = 0.05 and Gly = 0.05) and an increase in the levels of 9 amino acids (in average 9.7 increase) (Fold >2 or Fold <0.5; t-test, P<0.05). The most relevant amino acids are Gln (20.3 fold), Glu (13.7 fold), Asp (8.8) and Asn (5.6) in the Ama^axe^. These results highlight the importance of these amino acids for amastigotes. While the requirement of Glu and Asp are slightly slower in comparison to the Pro^log^ the requirements of Gln and Asn were equivalent in both stages.

**Fig 6:**
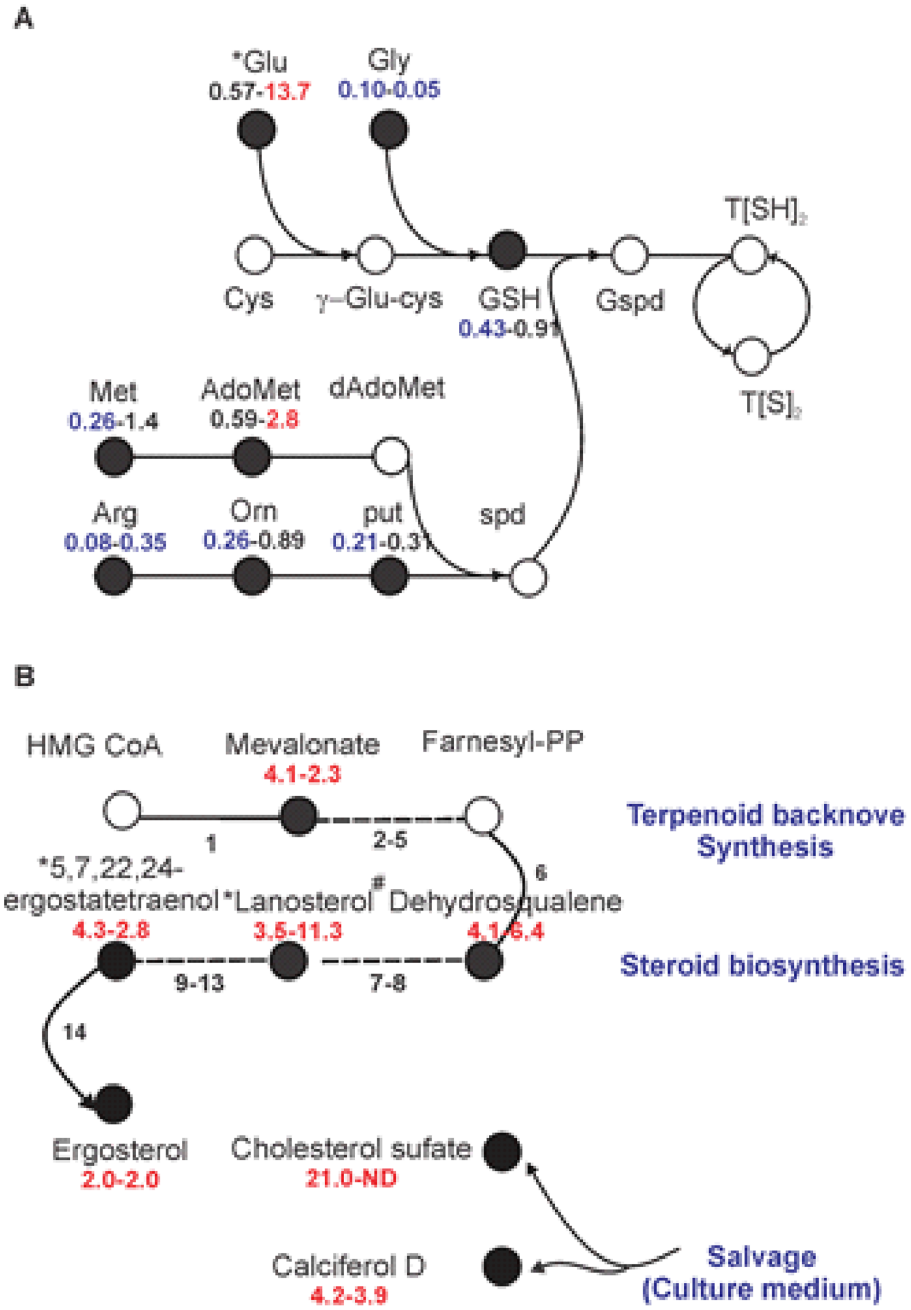
Polyamines and steroid levels in *L*. *(V*.*) braziliensis* Ama^axe^ in comparison to Pro^log^ and Pro^sta^. **A**, Schematic overview of polyamines pathway in trypanosomatids. Abbreviations are as follows: Orn, ornithine; Put, putrescine; Spd, spermidine; AdoMet, S-adenosylmethionine; dAdoMet, decarboxylated S-adenosylmethionine; GSH, reduced glutathione; T[S]_2_ oxidized trypanothione (trypanothion disulfide); T[SH]_2_ reduced trypanothione; Gspd, glutathionyl-spermidine. **B**, Schematic overview of terpenoid backbone synthesis and steroid biosynthesis, leading to the production of ergosterol. The dashed lines represent more than one reaction. Metabolites in empty circles were not detected in this study. The fold changes are shown below the name of each metabolite, the first number represents the signal intensity ratio for Ama^axe^/Pro^log^ and the second number represent the signal intensity ratio for Ama^axe^/ Pro^sta^. The metabolites with significant increased levels are shown in red (> 2 fold; t-test, P< 0.05), while metabolites with significant decreased levels are shown in blue (<0.5 fold; t-test, P< 0.05). The asterisk indicates that another isomer was detected for this metabolite. ^#^ Dehydrosqualene is not described in the *L*. *donovani* KEGG steroid synthesis, but is the results of linking 2 farnesyl-PP via presqualene-PP and can then be reduced to squalene. ND, No cholesterol sulfate was detected in stationary parasites.

Eight metabolites involved in the synthesis of polyamines were detected and six of them showed decreased levels (in average 0.22 fold) in Ama^axe^ in comparison to Pro^log^ (Fold <0.5; t-test, P<0.05), while the other two metabolites also followed the same trend (glutamate = 0.57, S-adenosylmethionine = 0.59) (Fig 6A). A similar trend was observed when comparing Pro^sta^ and Pro^log^, with decreased levels of 6 metabolites of this pathway (in average 0.24 fold) (Fold <0.5; t-test, P<0.05). Altogether this shows the lower level of polyamines in life stages characterized by lower/no cell proliferation.

The sterol and lipid composition were another interesting finding. The Ama^axe^ had increased levels of the endogenous ergosterol and its precursors of the terpenoid pathway (mevalonate) and steroid biosynthesis pathway (dehydrosqualene, lanosterol, ergostatetraenol), compared to both stages of the promastigotes (Fold > 2.0; t-test, P<0.05) (Fig 6B). Increased levels of exogenous sterols as cholesterol (21 fold) and calciferol D (4.2 fold) were also found in Ama^axe^ in comparison to Pro^log^ (Fig 6B).

**Fig 7:**
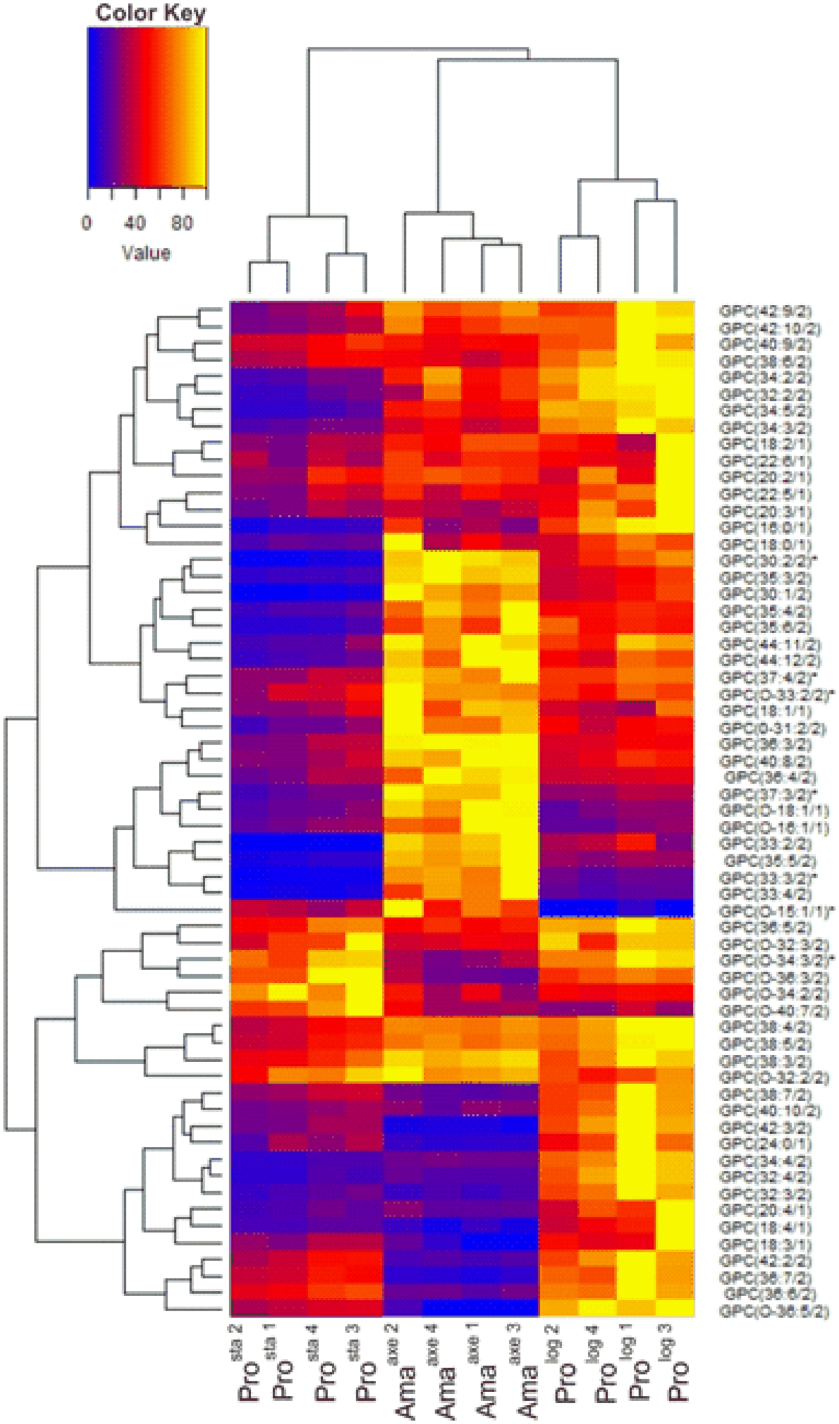
Metabolic profiles of GPCs in heatmap format. The samples are presented along the bottom (Pro^sta^, Ama^axe^, Pro^log^, the numbering represents the biological replicate). The branches on the top and on the left represent the dendrogram after hierarchical cluster analysis. For each metabolite, its abundance over the different biological replicates was rescaled between 0 (blue) and 100 (yellow). The corresponding fold changes between conditions are displayed in S4 Table. On the right, GPCs should be interpreted as follows: GPC (x:y/z), where x represents the number of carbons in the fatty acid side chain(s), y represents the number of double bonds, and z represents the number of side chains. The asterisk indicates that another isomer was detected for this metabolite (see S2 Table for more details).

Among phospholipids, we found a high number of glycerophosphocholines (GPC) and glycerophosphatidylethanolamines (GPE); the high diversity of these phospholipids is generated by the different length of their acyl groups and their different degree of unsaturation. Ama^axe^ showed different patterns in comparison to promastigotes, but following a different trend when considering Pro^log^ or Pro^sta^ (Fig 7 and S4 Table, S4 Fig and S5 Table). When compared to Pro^log^, Ama^axe^ showed 17 GPCs (out of 54), which were decreased (in average 0.20 fold), and 7 increased (in average 2.9 fold). The same trend was shown with GPEs (23) as Ama^axe^ in comparison to Pro^log^ had decreased levels of 7 GPEs (in average 0.30 fold), but increased levels of 4 of them (in average 2.8 fold). When Ama^axe^ were compared to Pro^sta^, 11 GPCs showed a decrease (in average 0.33 fold), while 23 increased (in average 3.92 fold). A same trend was observed for GPEs, with 4 decreased (in average 0.29 fold) and 10 increased (in average 5.17 fold).

## Discussion

Our study present cellular, molecular and metabolic evidences that *L*. *(V*.*) braziliensis in vitro* amastigotes are a life stage with a negligible rate of proliferation, a lower capacity of biosynthesis, a reduced bio-energetic level and a strongly altered metabolism.

The multiplication of *in vitro* promastigotes, with a doubling time of 11.8 hours, contrasts with the restricted growth of axenic amastigotes, the stable percentage of infected macrophages and the stable number of amastigotes per infected macrophage over 5 days of *in vitro* infection. The same behavior has been reported *in vitro* in *L*. *(L*.*) donovani*, *L*. *(L*.*) infantum* and *L*. *(L*.*) chagasi* in bone marrow macrophages [25,26] and *in vitro/in vivo* in *L*. *(L*.*) mexicana* [8]. The low proliferation could be associated with parasites (i) replicating and dying at the same rate or (ii) non-replicating and non-dying. The cell viability assay here applied did not highlight significant death rate among amastigotes, thus indirectly supporting the second explanation. Cell proliferation is an energetically expensive process, that requires DNA, RNA and protein synthesis. The translation itself is the most expensive process during the cell growth and it consumes about 70% of the ATP content of cells [27,28]. Therefore, the decrease in proliferation should be related to the observed decrease of the intracellular ATP levels, the protein synthesis machinery and translation process itself. This was confirmed by a series of observations.

We showed a significant decrease of ATP in Pro^sta^, Ama^axe^ and Ama^int^; while ADP levels were rather stable. Extrapolating the metabolomics results obtained on Ama^axe^ (lower AMP), this would suggest that the adenylate energy charge (AEC) defined by the equation [ATP+ (ADP/2)]/(ATP+ADP+AMP) is decreased in amastigotes. Similarly, *L*.*(L) donovani* amastigotes also have decreased levels of ATP and AEC, consequently to an attenuated oxidative phosphorylation [29]. As such, the decreased levels of ATP here observed could also be related with the significant decrease of kDNA minicircles we observed here in Ama^int^: kDNA minicircles are known to encode guide RNAs involved in the edition and regulation of kDNA maxicircle transcripts encoding several subunits of the respiratory complexes of the inner mitochondrial membrane [30,31].

Another relevant feature of cells with low proliferation is the reduction in the level of transcription of ribosomal components and proteins related with their biogenesis. This was clearly demonstrated in *Mycobacterium bovis* (3880 and 687 ribosomes per cell in strains with a generation time of 23 and 69 hrs respectively) [32] and in quiescent cells of *Schizosaccharomyces pombe* [33–35]. We showed here that Ama^axe^ of *L*. *(V) braziliensis* were characterized by a drop (in comparison with Pro^log^) in the amount of 18S and 28S rRNA (in average to 15% of the Pro^log^ amount) together with the corresponding ribosomal proteins RPS15 and RPL19. Altogether, our findings support the occurrence of a lower number of ribosomes in amastigotes that might be associated with the observed decrease (about 40%) in total protein content. Similarly, in species like *L*. *(L*.*) infantum* and *L*. *(L*.*) donovani*, differentiation to Ama^axe^ was coupled with a reduction in the amount of polysomes and a decrease of 50% in the translation [36,37]. Moreover, proteomic analysis of *L*. *(L) donovani* during the differentiation from late logarithmic promastigotes to amastigotes showed a progressive decrease in the levels of ribosomal proteins, translation factors and tRNA synthetases [38].

The data mentioned above support the hypothesis that *L*. *(V*.*) braziliensis* amastigotes are metabolically less active than proliferative promastigotes. In order to further document this hypothesis, we performed untargeted metabolomics on Pro^log^, Pro^sta^ and Ama^axe^. The parasite load is too low upon infection of mice with *L*. *(L*.*) braziliensi*s [39–41] to obtain the quantity of cells needed for metabolomics. Therefore, we focused on Ama^axe^, a stage that is generally considered as a model of intracellular amastigotes, at molecular, metabolic and ultrastructural levels [9,42,43]. We found clear differences in terms of amino acids, polyamines, GPLs and sterols. Firstly, the significant drop in amino acid levels in Ama^axe^ is in agreement with a lower biosynthetic activity. Similarly, a stringent metabolic response has been reported *in L*. *(L*.*) mexicana* amastigotes, with a strong decrease in the uptake and consumption rate of glucose and amino acids [9]. Second, amastigotes showed a downregulation in the synthesis of polyamines (8 detected metabolites), involved in several cellular functions such as cell proliferation and response to cellular stress [44]. On one hand, since one of the main functions of polyamines is to encompass DNA synthesis, thus the decrease is in agreement with a lower proliferation rate in amastigotes [45]. On the other hand, polyamines are involved in trypanothione synthesis that is required to survive oxidative burst in macrophages. We could not detect this metabolite in our study, but our results might predict a decrease in trypanothione production. Further work would be required to know the consequences of downregulations of polyamines in the context of resistance to stress. Thirdly, the major changes in GPLs could be related to developmental changes in the *de novo* synthesis of phospholipids via the Kennedy pathway and/or salvage from the medium [46–48]. Fourthly, an overall increase in metabolites related to the steroid biosynthesis pathway and the incorporation of cholesterol was found in Ama^axe^. Two sterols deserve particular attention here, both being increased in Ama^axe^ compared to Pro^log^ and Pro^sta:^ (i) the endogenous ergosterol and its precursors of the terpenoid and steroid biosynthesis pathways and (ii) the exogenous cholesterol. Previous studies showed an increased level of cholesterol in amastigotes of *Trypanosoma cruzi*, *L*. *major* and *L*. *mexicana* [49–51]. Most sterols are located in the cell membrane where they play an important role in membrane fluidity, hydrophobic thickness and permeability [52,53]. Moreover, ergosterol was shown to protect *L*. *(L*.*) donovani* against oxidative stress [54] and sterols do constitute a carbon and energy source [55].

We conclude that the *in vitro* low-proliferating amastigotes of *L*. *(V*.*) braziliensis* are endowed with several features expected for a quiescent stage. However, further work should include a direct demonstration of their low/no-replication as well as the heterogeneity of the phenomenon: this could be done at single cell level by BrdU labeling [10]. This should be complemented by *in vivo* work, especially in the context of oxidative burst and chemotherapy. Such studies would contribute to a better understanding of the persistence of infection that has been reported extensively in Leishmaniasis [6,56–59].

## Supporting information

S1 Table Primers and conditions for qPCR assays. (DOCX)

S2 Table List of 186 biological analytes. List of 186 putatively identified metabolites with the following information for each compound: (A) detection modus (positive or negative modus); (B) database in which the metabolite was detected (ITM = in-house *Leishmania* database based on LeishCyc and further completed with identifications from Lipid MAPS); (C) detected mass; (D) chromatographic retention time; (E) converted chromatographic retention time; (F) retention time of external standards; (G) putative metabolite identification; (H) compound class (NA: not assigned); (I) compound subclass (NA: not assigned); (J-Y) signal intensity in the four biological replicates of the samples (Pro^log^, Pro^sta^, Ama^axe^) (each line is color-coded); (V-X) average intensity of each sample; (Y-Z) ratio of average signal intensity of Ama^axe^ versus average signal intensity of Pro^log^ followed by p value of a t-test assuming unequal variance rank; (AA-AB) ratio of average signal intensity of Ama^axe^ versus average signal intensity of Pro^sta^ followed by p value of a t-test assuming unequal variance rank; (AC-AD) ratio of average signal intensity of Pro^log^ versus average signal intensity of Pro^sta^ followed by p value of a t-test assuming unequal variance rank. Ratios in bold and red were metabolites with a fold change higher than 2 and with p < 0.05, ratios in bold and blue were metabolites with a fold change lower than 0.5 with p < 0.05. Ratios in bold had a significant corresponding p value (t-test assuming unequal variance) but no significant fold change (0.5 < x < 2). KEGG: Kyoto Encyclopedia of Genes and Genomes (http://www.genome.jp/kegg); Lipidmaps: LIPID Metabolites and Pathways Strategy (http://www.lipidmaps.org). Glycerophospholipids (GPLs) should be interpreted as follows: GPL(x:y/z), where x represents the number of carbons in the fatty acid side chain(s), y represents the number of double bonds, and z represents the number of side chains.
(XLSX)

S3 Table Amino acid fold changes. Values in bold indicate that the fold change (FC) is significant (P<0.05). FCs >2 are marked red and FCs < 0.5 blue. Axe-A: Axenic amastigotes, Log-P: Logarithmic phase promastigotes, Sta-P: Logarithmic phase promastigotes. The asterisk indicates that another isomer was detected for this metabolite (see S2 Table).
(DOCX)

S4 Table Glycerophosphocholines fold changes. Values in bold indicate that the fold change (FC) is significant (P<0.05). FCs >2 are marked red and FCs < 0.5 blue. Axe-A: Axenic amastigotes, Log-P: Logarithmic phase promastigotes, Sta-P: Logarithmic phase promastigotes. The different GPCs should be interpreted as follows: GPC (x:y/z), where x represents the number of carbons in the fatty acid side chain(s), y represents the number of double bonds, and z represents the number of side chains. The asterisk indicates that another isomer was detected for this metabolite (see S2 Table).
(DOCX)

S5 Table Fold changes of GPEs, GPPs and GPIs. Values in bold indicate that the fold change (FC) is significant (P<0.05). FCs >2 are marked red and FCs < 0.5 blue. Axe-A: Axenic amastigotes, Log-P: Logarithmic phase promastigotes, Sta-P: Logarithmic phase promastigotes. The different GPX should be interpreted as follows: GPX (x:y/z), where x represents the number of carbons in the fatty acid side chain(s), y represents the number of double bonds, and z represents the number of side chains. GPE: glycerophosphoethanolamine; GPP: glycerophosphate, GPI: glycerophosphoinositol. The asterisk indicates that another isomer was detected for this metabolite (see S2 Table).
(DOCX)

S1 Fig Real time qPCR melting curves and standard curves for the quantification of 18S and 28S rRNA. A. Real time qPCR for 18S rRNA. The curve standard was generated with 1× 10^7^ to 1× 10^2^ plasmid copies (containing the *L*.*(V*.*) braziliensis* amplicon). The standard curve was characterized by a mean square error (MSE) ≤ 0.030, a slope of -3.45 (mean) ± 0.03 (standard deviation) indicating a high amplification efficiency (≥ 1.94) B. Real time qPCR for 28S α rRNA. The standard curve was generated with 1× 10^8^ to 1× 10^3^ plasmid copies. The dynamic range of the 28S α rRNA-qPCR assay encompassed at least 6 orders of magnitude (1 × 10^9^ to 1 × 10^3^ plasmid copies /reaction). The standard curve was characterized by a mean square error (MSE) ≤ 0.054, a slope of -3.48 ± 0.05 indicating a high amplification efficiency (≥ 1.92). Under the standardized conditions both assays did not amplify genomic DNA or cDNA of mouse.
(TIF)

S2 Fig Growth kinetics of intracellular amastigotes of a clinical isolate of *L*. *braziliensis* (MHOM/PE/03/PER206). A ratio of 8 amastigotes per macrophage was used. The percentage of infected macrophages and the amastigotes per macrophage were counted 36 48 and 72 hrs. post infection.
(TIF)

S3 Fig Evaluation of the viability of the harvested parasites. The parasites were stained simultaneously with the permeable fluorofore DyeCycle Ruby and the non-permeable fluorofore NucGreen, which stain the DNA of live and dead parasites, respectively. A control of unstained Pro^log^ and a control of dead parasites (stationary promastigotes at day 10) are included. Ama^axe^ and the Ama^int^ were purified by a gradient of percoll before the analysis. For each stage, one sample representative of three biological replicates is shown.
(TIF)

S4 Fig Metabolic profiles of GPEs, GPPs and GPIs in heatmap format. The samples are presented below the heatmap (Pro^sta^, Ama^axe^, Pro^log^, the numbering represents the biological replicate). For each metabolite, its abundance over the different biological replicates was rescaled between 0 (blue) and 100 (yellow). The corresponding fold changes between conditions are displayed in S5 Table. The branches on the top and on the left represent the dendrogram after Hierarchical Cluster Analysis. On the right, the different GPLs should be interpreted as follows: GPL (x:y/z), where x represents the number of carbons in the fatty acid side chain(s), y represents the number of double bonds, and z represents the number of side chains. GPE: glycerophosphoethanolamine; GPP: glycerophosphate, GPI: glycerophosphoinositol. The asterisk indicates that another isomer was detected for this metabolite (see S2 Table).
(TIF)
