# Supplementary material for: Macromolecular biosynthetic parameters and metabolic profile in different life stages of Leishmania braziliensis: Amastigotes as a functionally less active stage
Source: PLoS One. 2017 Jul 25;12(7):e0180532. doi: 10.1371/journal.pone.0180532 (PMC5526552; doi:10.1371/journal.pone.0180532)
Supplement: S1 Table — (DOCX) [file pone.0180532.s001.docx]

**Table S1. Primers and conditions for qPCR assays.**

| **Primer** | **Sequence** |  |  | **T ° annealing**  **Cycles** | **qPCR**  **Assay** | **Amplicon length (bp)** |
| --- | --- | --- | --- | --- | --- | --- |
| 18S F | 5' CATCAAACTGTGCCGATTACGTCC 3' | | | 65 ^o^C | qPCR-18S | 114 |
| 18S R | 5' GAACTTTCGGGCGGATAAAACACC 3' | | | 35 x |  |  |
| 28S α F | 5' CTAAGATGGACCGGCCTCTAGTGCA 3' | | | 69 ^o^C | qPCR-28S α | 195 |
| 28S α R | 5' AACTGCCGCCGACACACCTTCACT 3’ | | | 32 x |  |  |
