# Supplementary material for: Macromolecular biosynthetic parameters and metabolic profile in different life stages of Leishmania braziliensis: Amastigotes as a functionally less active stage
Source: PLoS One. 2017 Jul 25;12(7):e0180532. doi: 10.1371/journal.pone.0180532 (PMC5526552; doi:10.1371/journal.pone.0180532)
Supplement: S3 Table — Values in bold indicate that the fold change (FC) is significant (P<0.05). FCs >2 are marked red and FCs < 0.5 blue. Axe-A: Axenic amastigotes, Log-P: Logarithmic phase promastigotes, Sta-P: Logarithmic phase promastigotes. The asterisk indicates that another isomer was detected for this metabolite (see S2 Table). (DOCX) [file pone.0180532.s003.docx]

**Table S3. Amino acid fold changes**

| Metabolite | Axe-A/Log-P FC | Axe-A/Sta-P  FC | Log-P/Sta-P  FC |
| --- | --- | --- | --- |
| proline | **0.01** | **1.94** | **157.08** |
| citrulline | **0.03** | **0.15** | **4.66** |
| leucine* | **0.04** | **3.17** | **76.59** |
| lysine | **0.05** | **0.31** | **6.24** |
| Threonine* | **0.07** | 1.09 | **16.36** |
| arginine | **0.08** | **0.35** | **4.34** |
| glycine | **0.10** | **0.05** | **0.53** |
| serine | **0.17** | **2.65** | **15.77** |
| cystathionine | **0.24** | **3.62** | **14.96** |
| methionine | **0.26** | **1.39** | **5.31** |
| proline betaine | **0.31** | **0.05** | **0.17** |
| aspartic acid | **0.38** | **8.79** | **23.27** |
| tyrosine | **0.46** | **0.54** | 1.16 |
| alanine | **0.47** | **14.58** | **30.84** |
| phenylalanine | **0.51** | 1.00 | **1.98** |
| glutamic acid* | **0.57** | **13.74** | **24.21** |
| asparagine | 0.87 | **5.56** | **6.35** |
| glutamine | 0.93 | **20.29** | **21.76** |
| trimethyl-lysine | 1.10 | **0.08** | **0.07** |
| tryptophan | **1.72** | **1.60** | 0.93 |
| histidine | **4.44** | **8.73** | **1.97** |
