# Supplementary material for: Macromolecular biosynthetic parameters and metabolic profile in different life stages of Leishmania braziliensis: Amastigotes as a functionally less active stage
Source: PLoS One. 2017 Jul 25;12(7):e0180532. doi: 10.1371/journal.pone.0180532 (PMC5526552; doi:10.1371/journal.pone.0180532)
Supplement: S4 Table — Values in bold indicate that the fold change (FC) is significant (P<0.05). FCs >2 are marked red and FCs < 0.5 blue. Axe-A: Axenic amastigotes, Log-P: Logarithmic phase promastigotes, Sta-P: Logarithmic phase promastigotes. The different GPCs should be interpreted as follows: GPC (x:y/z), where x represents the number of carbons in the fatty acid side chain(s), y represents the number of double bonds, and z represents the number of side chains. The asterisk indicates that another isomer was detected for this metabolite (see S2 Table). (DOCX) [file pone.0180532.s004.docx]

**Table S4. Glycerophosphocholines fold changes**.

| Metabolite | Axe-A/Log-P | Axe-A/Sta-P | Log-P/Sta-P |
| --- | --- | --- | --- |
| GPC(35:5/2) | **2.91** | **11.59** | **3.99** |
| GPC(36:4/2) | **2.13** | **3.11** | **1.46** |
| GPC(36:6/2) | **0.25** | **0.38** | **1.51** |
| GPC(0-31:2/2) | **1.71** | **3.92** | **2.29** |
| GPC(16:0/1) | **0.43** | **4.16** | **9.73** |
| GPC(18:0/1) | 0.94 | 2.97 | **3.18** |
| GPC(18:1/1) | **1.86** | **2.58** | 1.39 |
| GPC(18:2/1) | 0.98 | **1.93** | 1.96 |
| GPC(18:3/1) | **0.12** | **0.23** | 1.91 |
| GPC(18:4/1) | **0.13** | **0.50** | **3.68** |
| GPC(20:2/1) | 0.95 | 1.48 | 1.56 |
| GPC(20:3/1) | **0.50** | 1.32 | **2.63** |
| GPC(20:4/1) | **0.31** | 1.19 | **3.83** |
| GPC(22:5/1) | 0.68 | **1.50** | **2.20** |
| GPC(22:6/1) | 0.92 | **1.71** | 1.85 |
| GPC(24:0/1) | **0.15** | **0.36** | **2.45** |
| GPC(30:1/2) | **1.69** | **37.99** | **22.50** |
| GPC(30:2/2)* | **1.52** | **24.73** | **16.24** |
| GPC(32:2/2) | **0.60** | **3.18** | **5.29** |
| GPC(32:3/2) | **0.17** | 0.81 | **4.76** |
| GPC(32:4/2) | **0.21** | 1.42 | **6.80** |
| GPC(33:2/2) | **2.40** | **absent in STAT** | **absent in STAT** |
| GPC(33:3/2)* | **4.12** | **28.21** | **6.84** |
| GPC(33:4/2) | **4.27** | **19.01** | **4.45** |
| GPC(34:2/2) | **0.68** | **3.14** | **4.65** |
| GPC(34:3/2) | **0.50** | **2.11** | **4.24** |
| GPC(34:4/2) | **0.26** | **1.70** | **6.40** |
| GPC(34:5/2) | **0.58** | **4.01** | **6.85** |
| GPC(35:3/2) | **1.90** | **7.61** | **4.00** |
| GPC(35:4/2) | **1.53** | **5.14** | **3.36** |
| GPC(35:6/2) | 1.29 | **7.06** | **5.46** |
| GPC(36:3/2) | **2.14** | **3.22** | **1.51** |
| GPC(36:5/2) | **0.61** | 0.85 | **1.40** |
| GPC(36:7/2) | **0.11** | **0.18** | **1.71** |
| GPC(37:3/2)* | **2.84** | **4.70** | **1.66** |
| GPC(37:4/2)* | **1.32** | **2.64** | **2.00** |
| GPC(38:3/2) | 1.08 | **1.61** | **1.49** |
| GPC(38:4/2) | 0.86 | **1.66** | **1.94** |
| GPC(38:5/2) | 0.86 | **1.56** | **1.82** |
| GPC(38:6/2) | **0.52** | 1.05 | **2.03** |
| GPC(38:7/2) | **0.26** | **0.56** | **2.21** |
| GPC(40:10/2) | **0.32** | 0.92 | **2.88** |
| GPC(40:8/2) | **1.86** | **2.98** | **1.61** |
| GPC(40:9/2) | **0.65** | 1.06 | **1.64** |
| GPC(42:10/2) | 0.78 | **2.22** | **2.86** |
| GPC(42:2/2) | **0.19** | **0.33** | **1.80** |
| GPC(42:3/2) | **0.07** | **0.20** | **2.94** |
| GPC(42:9/2) | 0.94 | **2.27** | **2.42** |
| GPC(44:11/2) | 1.28 | **4.75** | **3.71** |
| GPC(44:12/2) | **1.56** | **5.59** | **3.58** |
| GPC(O-15:1/1)* | **28.04** | **2.01** | **0.07** |
| GPC(O-16:1/1) | **3.50** | **3.37** | 0.96 |
| GPC(O-18:1/1) | **3.60** | **4.28** | 1.19 |
| GPC(O-32:2/2) | **1.32** | 1.18 | 0.90 |
| GPC(O-32:3/2) | **0.55** | 0.67 | 1.22 |
| GPC(O-33:2/2)* | **1.40** | **2.04** | **1.46** |
| GPC(O-34:2/2) | 0.80 | **0.46** | **0.57** |
| GPC(O-34:3/2)* | **0.36** | **0.39** | 1.09 |
| GPC(O-36:3/2) | **0.37** | **0.30** | 0.82 |
| GPC(O-36:5/2) | **0.04** | **0.09** | **2.34** |
| GPC(O-40:7/2) | 1.18 | **0.48** | **0.41** |
