# Supplementary material for: Macromolecular biosynthetic parameters and metabolic profile in different life stages of Leishmania braziliensis: Amastigotes as a functionally less active stage
Source: PLoS One. 2017 Jul 25;12(7):e0180532. doi: 10.1371/journal.pone.0180532 (PMC5526552; doi:10.1371/journal.pone.0180532)
Supplement: S5 Table — Values in bold indicate that the fold change (FC) is significant (P<0.05). FCs >2 are marked red and FCs < 0.5 blue. Axe-A: Axenic amastigotes, Log-P: Logarithmic phase promastigotes, Sta-P: Logarithmic phase promastigotes. The different GPX should be interpreted as follows: GPX (x:y/z), where x represents the number of carbons in the fatty acid side chain(s), y represents the number of double bonds, and z represents the number of side chains. GPE: glycerophosphoethanolamine; GPP: glycerophosphate, GPI: glycerophosphoinositol. The asterisk indicates that another isomer was detected for this metabolite (see S2 Table). (DOCX) [file pone.0180532.s005.docx]

**Table S5. Fold changes of GPEs, GPPs and GPIs**.

| Metabolite | Axe-A/Log-P | Axe-A/Sta-P | Log-P/Sta-P |
| --- | --- | --- | --- |
| glycerophosphoinositol | 0.72477 | **1.86971** | 2.579729 |
| GPE(18:1/1)* | **2.112163** | **3.61126** | **1.709745** |
| GPE(18:2/1) | 0.923795 | 1.025578 | 1.110179 |
| GPE(35:2/2) | **0.61644** | **2.434522** | **3.949326** |
| GPE(35:3/2)* | **0.179874** | **0.651968** | **3.624582** |
| GPE(35:4/2) | **0.220978** | 1.327841 | **6.008919** |
| GPE(36:4/2) | **3.445848** | **13.87519** | **4.026639** |
| GPE(36:5/2) | 1.100795 | **6.271079** | **5.696863** |
| GPE(37:3/2) | **0.466069** | **1.443164** | **3.096462** |
| GPE(37:4/2) | **0.297104** | **1.486642** | **5.00377** |
| GPE(38:3/2) | **2.147225** | **5.158344** | **2.402331** |
| GPE(38:4/2)* | **1.693511** | **4.760886** | **2.811252** |
| GPE(38:5/2) | **1.611253** | **5.185528** | **3.21832** |
| GPE(38:6/2) | 1.148725 | **6.157829** | **5.360576** |
| GPE(39:2/2) | **1.639637** | **2.174416** | **1.326157** |
| GPE(39:3/2) | **2.047314** | **2.179521** | 1.064576 |
| GPE(39:4/2) | **1.907087** | **1.994229** | 1.045694 |
| GPE(39:5/2) | **0.558153** | **0.567283** | 1.016357 |
| GPE(39:6/2) | **0.233747** | **0.273159** | 1.168606 |
| GPE(40:8/2) | **3.810532** | **34.2121** | **8.9783** |
| GPE(41:3/2) | 1.077904 | 1.174957 | 1.090039 |
| GPE(41:4/2) | 0.890557 | 1.091611 | 1.225763 |
| GPE(41:6/2) | **0.522075** | **0.723996** | **1.386766** |
| GPE(41:7/2) | **0.236478** | **0.399202** | **1.688114** |
| GPE(O-34:3/2) | **0.413804** | **0.308431** | **0.745355** |
| GPE(O-36:4/2) | **0.368586** | **0.24081** | **0.653334** |
| GPI(36:2/2) | 1.295179 | **6.357144** | **4.908313** |
| GPI(36:3/2) | 1.101671 | **3.336321** | **3.028418** |
| GPP(43:6/2) | 0.876842 | 1.079127 | **1.230697** |
| MMGPE(36:2/2) | **0.642709** | **2.096162** | **3.26145** |
