# Supplementary figures and images for: Macromolecular biosynthetic parameters and metabolic profile in different life stages of Leishmania braziliensis: Amastigotes as a functionally less active stage
Source: PLoS One. 2017 Jul 25;12(7):e0180532. doi: 10.1371/journal.pone.0180532 (PMC5526552; doi:10.1371/journal.pone.0180532)

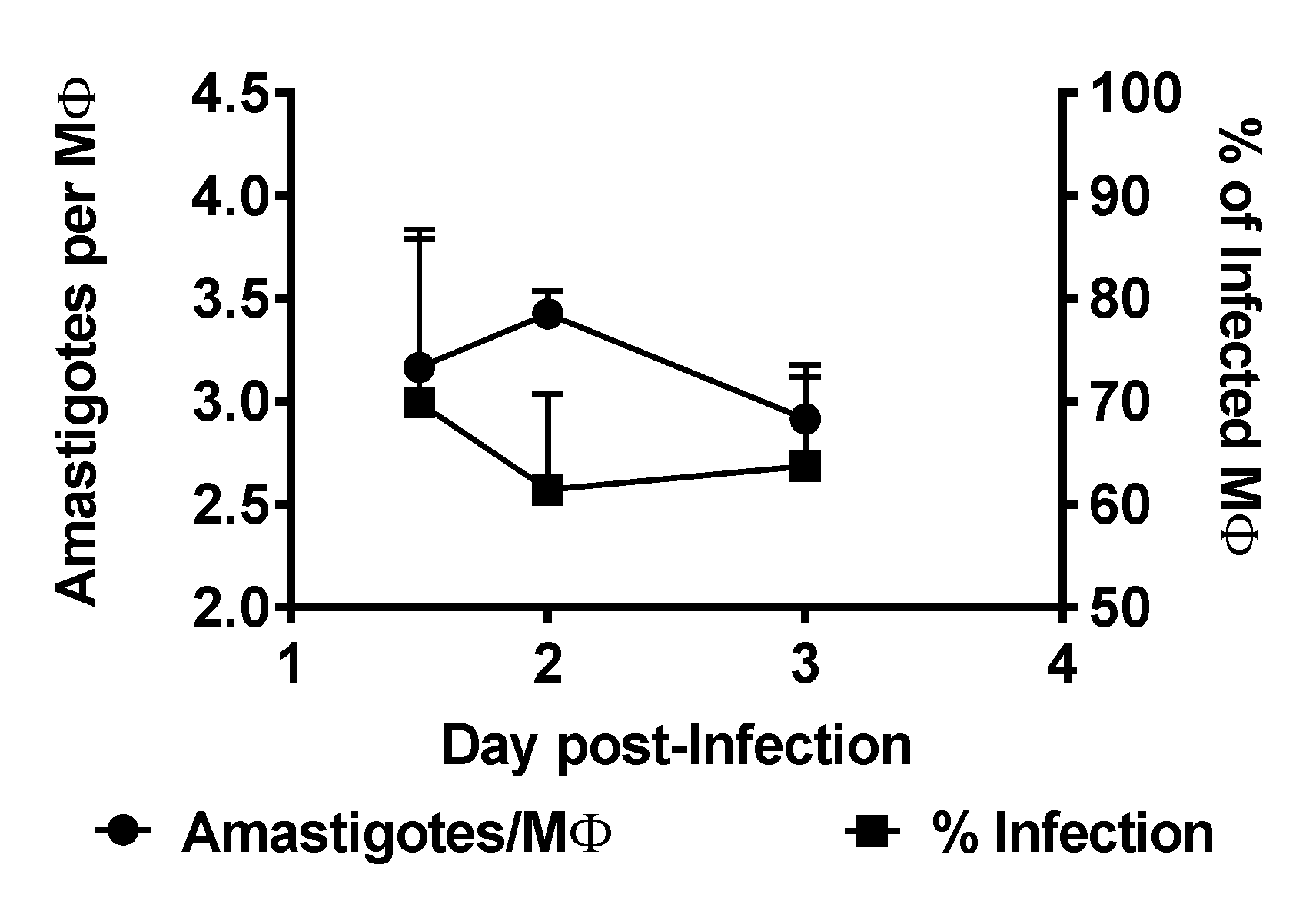

Supplement: S2 Fig — A ratio of 8 amastigotes per macrophage was used. The percentage of infected macrophages and the amastigotes per macrophage were counted 36 48 and 72 hrs. post infection. (TIF) [file pone.0180532.s007.tif]
